# The Role of Aerobic Fitness in Cortical Thickness and Mathematics Achievement in Preadolescent Children

**DOI:** 10.1371/journal.pone.0134115

**Published:** 2015-08-12

**Authors:** Laura Chaddock-Heyman, Kirk I. Erickson, Caitlin Kienzler, Matthew King, Matthew B. Pontifex, Lauren B. Raine, Charles H. Hillman, Arthur F. Kramer

**Affiliations:** 1 Beckman Institute, University of Illinois at Urbana-Champaign, Urbana, Illinois, United States of America; 2 Department of Psychology, University of Illinois at Urbana-Champaign, Champaign, Illinois, United States of America; 3 Department of Psychology, University of Pittsburgh, Pittsburgh, Pennsylvania, United States of America; 4 Department of Kinesiology, Michigan State University, East Lansing, Michigan, United States of America; 5 Department of Kinesiology & Community Health, University of Illinois at Urbana-Champaign, Urbana, Illinois, United States of America; University of Montreal, CANADA

## Abstract

Growing evidence suggests that aerobic fitness benefits the brain and cognition during childhood. The present study is the first to explore cortical brain structure of higher fit and lower fit 9- and 10-year-old children, and how aerobic fitness and cortical thickness relate to academic achievement. We demonstrate that higher fit children (>70th percentile VO_2max_) showed decreased gray matter thickness in superior frontal cortex, superior temporal areas, and lateral occipital cortex, coupled with better mathematics achievement, compared to lower fit children (<30th percentile VO_2max_). Furthermore, cortical gray matter thinning in anterior and superior frontal areas was associated with superior arithmetic performance. Together, these data add to our knowledge of the biological markers of school achievement, particularly mathematics achievement, and raise the possibility that individual differences in aerobic fitness play an important role in cortical gray matter thinning during brain maturation. The establishment of predictors of academic performance is key to helping educators focus on interventions to maximize learning and success across the lifespan.

## Introduction

Aerobic fitness and physical activity are beneficial to cognitive and brain health during development (see [1] for a review). Higher levels of aerobic fitness during childhood are associated with superior cognitive control, memory [2–8], and academic achievement [9,10]. Growing evidence suggests that these aerobic fitness differences in cognition and academics have a biological basis in the brain. In particular, higher fit children have larger structural brain volumes in the hippocampus and dorsal striatum, two subcortical regions critical for memory and learning [3,4], as well as more efficient brain activation patterns (via functional magnetic resonance imaging [fMRI] and event-related potential [ERP] measures) during attentional and interference control tasks [11–12; 5–8], relative to lower fit peers.

It is possible that aerobic fitness during childhood also influences the structure of cortical systems (as found in older adults, e.g., [13–14]), which may play a role in cognition and school performance. Cortical structure can be measured by determining cortical thickness, calculated by generating models of gray/white matter boundaries and pial surfaces, and calculating the distance between these two surfaces [15–18]. Normative maturation of cortical thickness provides a context to formulate predictions about how aerobic fitness might influence this brain measure in children. One longitudinal study scanned 45 children every 2 years from age 5 to 11 and demonstrated cortical thinning with development in dorsolateral frontal cortex, occipital-parietal areas, and anterior and posterior/inferior temporal regions (with rates of loss of approximately 0.1–0.3 mm per year) [19]. Furthermore, cortical thinning in the dorsal frontal and parietal regions was correlated with improved performance on a test of verbal intellectual functioning (vocabulary test of the Wechsler Intelligence Scale) [19]. In fact, research suggests that gray matter loss occurs as part of the sculpting of the brain into the fully functioning adult nervous system [19–20].

Higher levels of aerobic fitness are also known to predict better academic performance (e.g., mathematics, reading, English) during childhood [9–10; 21–22], and significant improvements in scholastic performance are associated with increased participation in physical activity during the school day [23–24]. However, little is known about the neural markers for academic success. In terms of neuroelectric indices, the P3 ERP component, reflective of attentional processes involved in stimulus evaluation and inhibition, has been suggested as a marker of reading and arithmetic achievement during childhood [25]. Here, we are the first to examine whether brain structural differences in higher and lower fit children relate to academic achievement. Understanding predictors of academic success, such as aerobic fitness and brain structure, has important implications, as standardized test performance can determine funding and effectiveness of educational programs as well as forecast a student's future scholastic success [26–27].

Given evidence that aerobic fitness is associated with specific measures of brain health and cognition during child development, we predicted that individual differences in aerobic fitness would be associated with cortical thickness, which would in turn be related to academic performance. Specifically, because cortical thinning is associated with brain development and maturation, we predicted that higher fit 9- and 10-year-old children would show decreased cortical thickness across the cortex, which would relate to better performance on the Wide Range Achievement Test (WRAT-3) of reading, spelling, and arithmetic achievement, relative to lower fit children.

## Materials and Methods

### Participants

Our study was reviewed and approved by the Institutional Review Board of the University of Illinois at Urbana-Champaign. Preadolescent 9- and 10-year-old children were recruited from East-Central Illinois. Children were screened for several factors that influence physical activity participation and cognitive function. The Kaufman Brief Intelligence Test (K-BIT) [28] was administered to each child to obtain a composite intelligence quotient (IQ) score including both crystallized and fluid intelligence measures. Participants were excluded if their scores were more than 1 standard deviation below the mean (85%). A guardian of the child also completed the Attention-Deficit Hyperactivity Disorder (ADHD) Rating Scale IV [29] to screen for the presence of attentional disorders. Participants were excluded if they scored above the 85th percentile. Pubertal timing was also assessed using a modified Tanner Staging System [30] with all participants at or below a score of 2 on a 5-point scale of developmental stages. In addition, socioeconomic status (SES) was determined by creating a trichotomous index based on three variables: participation in a free or reduced-price meal program at school, the highest level of education obtained by the child’s mother and father, and the number of parents who worked full-time [31].

Furthermore, eligible participants were required to (1) qualify as higher fit or lower fit (see Aerobic Fitness Assessment section), (2) demonstrate right handedness (as measured by the Edinburgh Handedness Questionnaire) [32], (3) report no adverse health conditions, physical incapacities, or neurological disorders, (4) report no use of medications that influenced central nervous system function, (5) successfully complete a mock Magnetic Resonance Imaging (MRI) session to screen for claustrophobia in an MRI machine, and (6) sign an informed assent approved by the University of Illinois at Urbana-Champaign. A legal guardian also provided written informed consent in accordance with the Institutional Review Board of the University of Illinois at Urbana-Champaign.

Forty-eight children were included in the analysis, including 24 higher fit participants (14 boys, 10 girls) and 24 lower fit participants (8 boys, 16 girls). Fifty-two children were eligible for the study and completed an MRI scan, and four children were excluded from analysis due to inaccurate gray-white tissue segmentation and motion noise in the reconstructed structural image.

### Aerobic Fitness Assessment

The aerobic fitness level of each child was determined by measuring maximal oxygen uptake (VO_2max_) using a computerized indirect calorimetry system (ParvoMedics True Max 2400) during a modified Balke protocol [33]. Specifically, participants ran on a motor-driven treadmill at a constant speed with increases in grade increments of 2.5% every 2 minutes until volitional exhaustion. Averages for oxygen uptake (VO_2_) and respiratory exchange ratio (RER; the ratio between carbon dioxide and oxygen) were assessed every 20 seconds. In addition, heart rate was measured throughout the fitness test (using a Polar heart rate monitor [Polar WearLink + 31, Polar Electro, Finland]), and ratings of perceived exertion were assessed every 2 minutes using the children’s OMNI scale [34].

VO_2max_ was defined when oxygen consumption remained at a steady state despite an increase in workload. Relative peak oxygen consumption was based upon maximal effort as evidenced by (1) a plateau in oxygen consumption corresponding to an increase of less than 2 mL/kg/min despite an increase in workload, (2) a peak heart rate greater than 185 beats per minute [33] accompanied by a heart rate plateau (i.e., an increase in work rate without a concomitant increase in heart rate) [35], (3) RER greater than 1.0 [36], and/or (4) ratings on the children’s OMNI scale of perceived exertion greater than 8 [34]. Relative peak oxygen consumption was expressed in mL/kg/min.

Aerobic fitness group assignments (i.e., higher fit and lower fit) were based on whether a child’s VO_2max_ value fell above the 70th percentile (for age and gender) or below the 30th percentile (for age and gender) according to normative data provided by Shvartz and Reibold [37]. Children who did not qualify as higher fit or lower fit were excluded. All participants were compensated $10/hour for the demographic and VO_2max_ protocol and $20 for participation in the MRI session.

### MR Imaging Protocol and Cortical Thickness Analysis

For all participants, high-resolution (1.3 mm×1.3 mm×1.3 mm) T1- weighted structural brain images were acquired using a 3D MPRAGE (Magnetization Prepared Rapid Gradient Echo Imaging) protocol with 144 contiguous axial slices, collected in ascending fashion parallel to the anterior and posterior commissures (echo time = 3.87ms, repetition time = 1800ms, field of view = 256mm, acquisition matrix 192mm×192mm, slice thickness = 1.3mm, and flip angle = 8°). All images were collected on a 3-T head-only Siemens Allegra MRI scanner.

Automated brain tissue segmentation and reconstruction of cortical surface models were performed on T1-weighted structural MRI images using the standard recon-all image processing pipeline in FreeSurfer, version 5.2.0 (Released May, 2013; http://surfer-nmr.mgh.harvard.edu/). FreeSurfer automatically labels cortical surfaces using a Desikan-Killiany cortical parcellation atlas (see 38 for the labeling protocol). That is, vertices along the cortical surface are assigned a given label based on local surface curvature, average convexity, prior label probabilities, and neighboring vertex labels [38–39]. Data from all participants were processed using the same Apple OSX 10.8 computer to ensure that the observed findings were not a function of differences in software, operating system, or hardware specifications [40].

**Fig 1:**
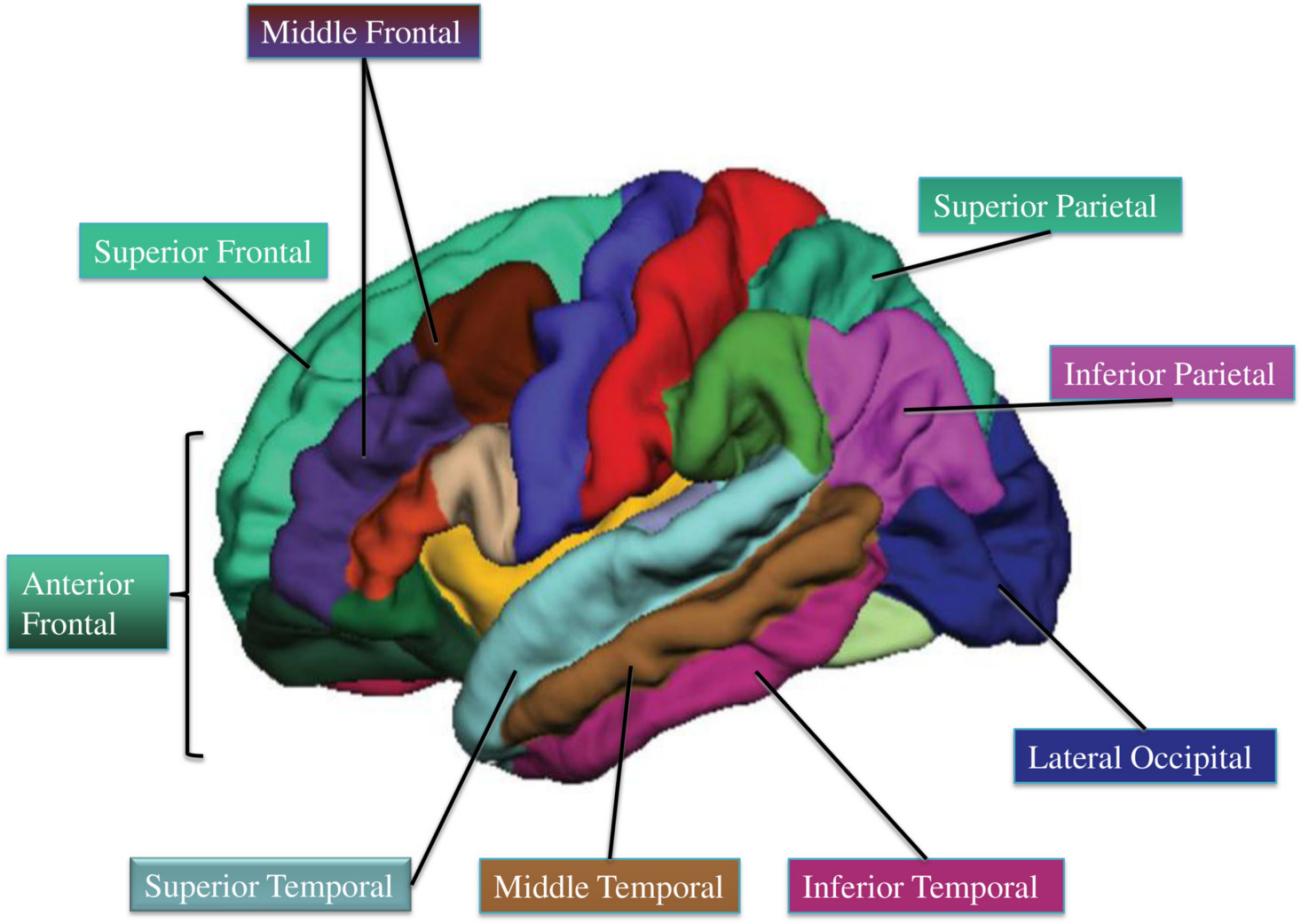
Cortical thickness regions of interest via Freesurfer (adapted from 43). Starred regions are areas in which higher fit children showed decreased cortical thickness compared to lower fit children.

Specifically, the following processing stream was applied to each participant’s structural image via FreeSurfer’s recon-all processing pipeline: (1) non-brain tissue removal, (2) Talairach transformation, (3) creation of representations of the gray/white matter boundaries [41–42], and (4) calculation of the cortical thickness as the distance between the gray/white matter boundary and the pial surface in all regions of interest [15]. Our *a priori* regions of interest included frontal (anterior, middle, superior), parietal (superior, inferior), temporal (superior, middle, inferior), and lateral occipital regions, as offered in FreeSurfer’s segmentation algorithms (**Fig 1, [43]**). These areas provide an exploratory analysis of the whole-brain and include regions of interest found to change with development [19]. Talairach transforms, skull stripping, gray–white tissue segmentation, and surface reconstructions were visually checked for errors (and, as noted, four children were excluded from analysis due to inaccurate gray-white tissue segmentation and motion noise in the reconstructed structural image).

### Wide Range Achievement Test (WRAT-3)

Academic achievement was assessed using the paper and pencil WRAT – 3rd edition (Wide Range, Inc., Wilmington, DE). The test battery included the content areas of reading (i.e., the number of words pronounced aloud correctly), spelling (i.e., the number of words spelled correctly), and arithmetic (i.e., the number of mathematical computations completed correctly). The WRAT-3 has been strongly correlated with the California Achievement Test–Form E and the Stanford Achievement Test [44].

### Statistical Analyses

Given the recruitment of higher and lower aerobic fitness groups, independent t-tests were conducted to compare demographic and fitness measures. We then performed a multivariate analysis of variance (MANOVA) to examine associations between aerobic fitness group (higher fit, lower fit) and cortical thickness in all areas of interest, across left and right hemispheres (Fig 1). Given a significant multivariate effect, secondary univariate ANOVAs were conducted to examine differences in cortical thickness between higher fit and lower fit children. Left and right thickness measures were averaged due to significant correlations between left and right thickness (all r>0.31, p<0.03) and no primary hypotheses about hemispheric differences as a function of aerobic fitness.

Additionally, independent t-tests were employed to compare WRAT-3 scores in higher fit and lower fit children. Pearson correlations were also conducted to determine associations between cortical thickness and academic achievement. The alpha level for all tests was set at *p* < .05.

## Results

Participant demographic and fitness data are provided in Table 1. Demographic variables (i.e., age, gender, IQ, ADHD, pubertal timing, SES) did not differ between fitness groups. Furthermore, consistent with our recruitment of extreme aerobic fitness groups, higher fit participants (M = 52.6 mL/kg/min, SD = 4.8 mL/kg/min) had higher VO_2max_ than lower fit children (M = 35.7 mL/kg/min, SD = 5.2 mL/kg/min) as revealed by an independent t-test (t (46) = 11.8, p<0.001).

**Table 1:** Participant mean demographic and fitness data (SD) by aerobic fitness group.

Variable	Lower Fit	Higher Fit
N	24 (16 girls)	24 (10 girls)
Age (years)	9.96 (0.64)	9.98 (0.61)
VO_2max_ (mL/kg/min)	35.65 (5.17) ^*^	52.64 (4.80) ^*^
VO_2max_ Percentile (%)	9.58 (5.55) ^*^	83.08 (4.84) ^*^
K-BIT^a^ Composite Score (IQ)	114.17 (15.24)	114.17 (7.63)
K-BIT^a^ Crystallized Score (Vocabulary)	108.79 (12.21)	109.04 (7.46)
K-BIT^a^ Fluid Score (Matrices)	116.58 (17.61)	116.42 (9.40)
ADHD^b^	5.96 (4.89)	7.33 (4.02)
Tanner^c^	1.63 (0.49)	1.67 (0.48)
SES^d^ (median)	2.71 (0.62)	2.63 (0.65)

^a^Kaufman Brief Intelligence Test [28].

^b^Scores on the *ADHD Rating Scale V* [29].

^c^Pubertal timing assessed using a modified Tanner Staging System (Tanner, 1962; 30].

^d^Socioeconomic Status. SES was determined by the creation of a trichotomous index based on three variables: child participation in a free or reduced-price lunch program at school, the highest level of education obtained by the child’s mother and father, and the number of parents who worked full-time [31].

*Significantly different at *p* < 0.05.

The overall multivariate test indicated a significant effect of aerobic fitness on cortical thickness (F (22, 27) = 2.41, p = 0.017). Next, univariate ANOVAs were performed to identify the specific cortical regions that contributed to the overall effect. Higher fit children showed decreased cortical thickness in superior frontal cortex (F (1, 46) = 4.80, p = 0.034), superior temporal cortex (F (1, 46) = 5.39, p = 0.025) and lateral occipital cortex (F (1, 46) = 5.67, p = 0.021), relative to lower fit children (Table 2). There was also specificity to the aerobic fitness differences, with some brain areas not showing aerobic fitness group differences in thickness, including the anterior frontal cortex (F (1, 46) = 2.33, p = 0.13), middle frontal cortex (F (1, 46) = 1.98, p = 0.17), middle temporal cortex F (1, 46) = 0.54, p = 0.47), inferior temporal cortex (F (1, 46) = 2.46, p = 0.12), superior parietal cortex (F (1, 46) = 1.21, p = 0.28), and inferior parietal areas (F (1, 46) = 0.63, p = 0.43) (Table 2). All cortical thickness values and effect sizes (Cohen’s d) are provided in Table 2.

**Table 2:** Cortical thickness (mean, standard deviation) as a function of aerobic fitness group.

Average Cortical Thickness	Lower Fit (M, SD)	Higher Fit (M, SD)	Effect size (Cohen’s d)
Anterior Frontal	3.55 (0.38)	3.38 (0.43)	0.420
Middle Frontal	3.41 (0.12)	3.35 (0.15)	0.515
Superior Frontal	3.85 (0.14)*	3.76 (0.15)*	0.620
Superior Parietal	2.93 (0.15)	2.89 (0.16)	0.258
Inferior Parietal	3.11 (0.18)	3.07 (0.18)	0.222
Superior Temporal	3.31 (0.19)*	3.17 (0.24)*	0.647
Middle Temporal	3.45 (0.16)	3.41 (0.16)	0.250
Inferior Temporal	3.28 (0.14)	3.21 (0.16)	0.466
Lateral Occipital	2.56 (0.19)*	2.46 (0.10)*	0.687

*p<0.05

**Fig 2:**
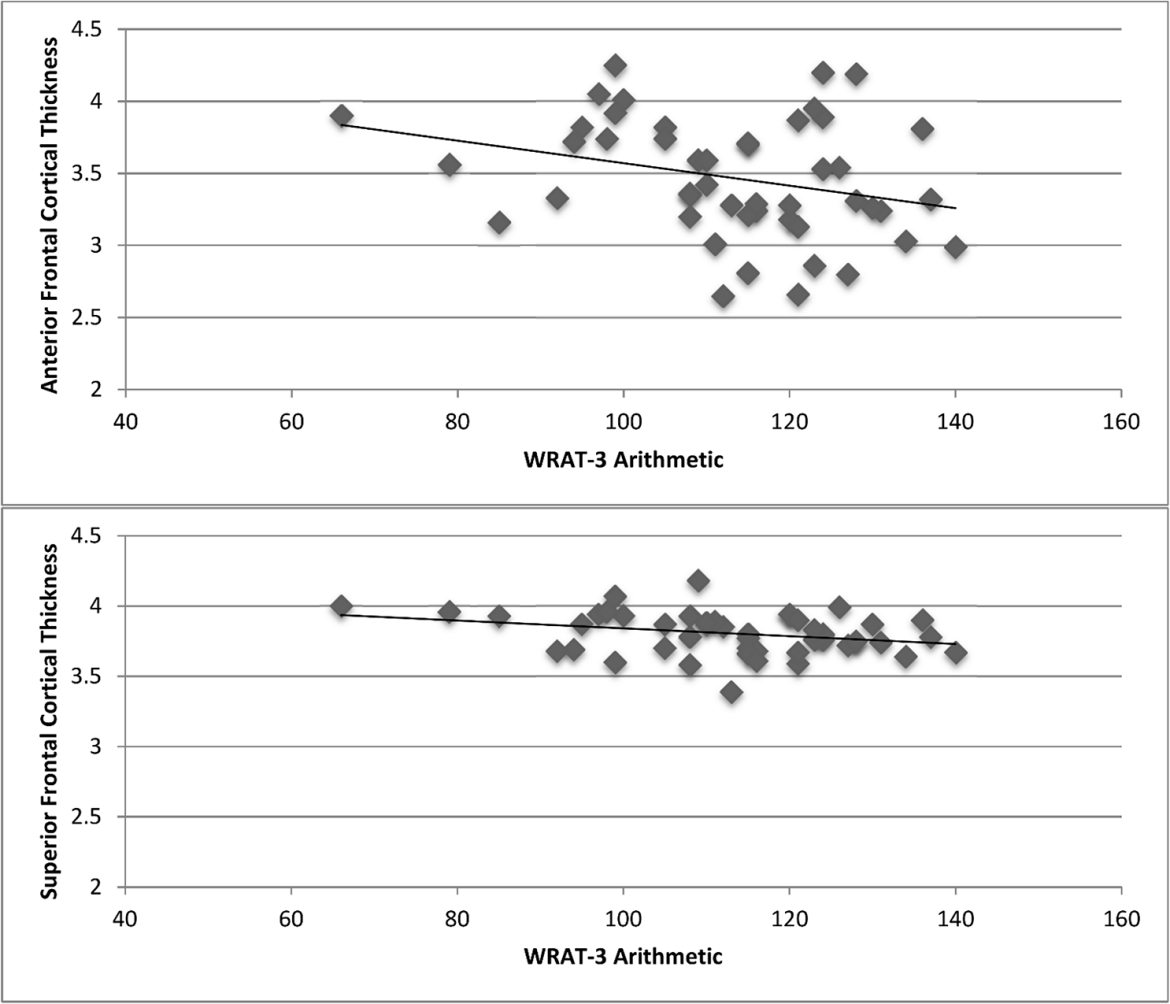
Significant associations between WRAT-3 mathematics achievement and cortical thickness.

Behaviorally, higher fit children showed superior mathematics achievement compared to lower fit children (t (46) = 1.98, p = 0.05) on the WRAT-3. No fitness differences were found for reading or spelling performance (t< 1.1, p>0.3). In addition, across all children, WRAT-3 arithmetic scores were negatively correlated with cortical thickness in anterior frontal cortex (r = -0.292, p = 0.04), and superior frontal cortex (r = -0.291, p = 0.04) (Table 3) (Fig 2).

**Table 3:** Pearson correlations (p-value) between cortical thickness and academic achievement in all children.

Average Cortical Thickness	WRAT-3 Reading	WRAT-3 Spelling	WRAT-3 Arithmetic
Anterior Frontal	-0.208 (0.16)	-0.143 (0.33)	-0.292 (0.04)*
Middle Frontal	0.032 (0.83)	0.070 (0.64)	-0.114 (0.44)
Superior Frontal	-0.045 (0.76)	0.013 (0.93)	-0.291 (0.04)*
Superior Parietal	0.114 (0.44)	0.093 (0.53)	0.012 (0.93)
Inferior Parietal	-0.110 (0.46)	-0.082 (0.58)	-0.231 (0.16)
Superior Temporal	-0.011 (0.94)	-0.096 (0.51)	-0.048 (0.75)
Middle Temporal	0.018 (0.90)	0.106 (0.48)	-0.227 (0.12)
Inferior Temporal	0.209 (0.15)	0.263 (0.07)	-0.206 (0.86)
Lateral Occipital	0.064 (0.67)	0.106 (0.47)	0.124 (0.40)

* p<0.05.

## Discussion

Consistent with predictions, our results demonstrate that higher fit 9- and 10-year-old children (>70th percentile VO_2max_) showed decreased gray matter thickness in superior frontal cortex, superior temporal areas, and lateral occipital cortex, coupled with better arithmetic performance on a standardized achievement test, compared to lower fit children (<30th percentile VO_2max_). Furthermore, cortical gray matter thinning in anterior and superior frontal areas was associated with superior mathematics achievement. Together, our data raise the possibility that individual differences in aerobic fitness play a role in childhood cortical gray matter structure important for scholastic success, particularly on mathematics tests. These novel results add to our understanding of developmental plasticity in brain and cognition as a function of aerobic fitness, as well as the neural correlates of performance on measures of importance in education.

Our results support and extend research on changes in cortical surface organization during child development [19] and across the lifespan [20]. That is, the brain areas that showed cortical thinning as a function of higher levels of aerobic fitness (i.e., frontal, temporal and occipital regions) are similar to developing brain areas that undergo significant cortical thinning between the ages of 5 and 11 (i.e., lateral frontal cortex, temporal regions, and occipital areas) [19]. Moreover, areas of the dorsal frontal cortex and superior frontal sulcus have demonstrated non-linear declines in gray matter density with age across the lifespan (age 7–60) [20]. Here, our data raise the possibility that individual differences in aerobic fitness may influence some areas that show significant changes in cortical thickness during development, and perhaps even across the lifespan. Although the methods for segmentation of cortical regions and calculation of gray matter thickness/density are not identical here and in previous work [19–20], it is possible that aerobic fitness is one predictor of the developmental trajectory of cortical structure in certain areas. It is important to note that we also demonstrate some specificity of the effects, as aerobic fitness was not associated with significant gray matter thickness differences in areas such as middle frontal, middle temporal and superior and inferior parietal cortex. Future work should employ whole-brain analyses corrected for multiple comparisons to continue to explore the specific effects of aerobic fitness on cortical brain structure during childhood.

We also found that decreased cortical gray matter thickness in anterior and superior frontal cortex predicted better performance on a paper and pencil test of mathematics achievement known to correlate with standardized achievement assessments in the classroom [44]. The results add to the biological markers for academic success [25], and also raise the possibility that fitness-related differences in cortical structure (in particular, the superior frontal cortex) have important scholastic implications (in particular, for mathematics achievement). Successful mathematics problem solving is said to involve working memory, the ability to hold relevant information in mind for efficient and effective comprehension [45–47] as well as inhibition, the ability to ignore irrelevant information [48]. Higher fit children have shown superior performance on cognitive control tasks that challenge working memory and inhibitory control [11–12, 5–6, 8], as well as superior performance on standardized tests of mathematics and reading [9–10], relative to lower fit children. Together, our study suggests that differences in cortical gray matter structure in frontal cortex may predict superior arithmetic performance in school, and aerobic fitness may be one pathway by which brain and cognition are enhanced during development.

Furthermore, it is interesting to note that we suggest a unique association among aerobic fitness, cortical thickness and specifically arithmetic achievement, rather than global scholastic success across reading, spelling and arithmetic. Future efforts should be directed toward determining additional neural biomarkers for scholastic success, and whether these biomarkers predict performance on select academic subjects, as suggested here, or whether they serve as a more global index of overall school performance. For instance, the P3 is suggested to be a marker of both reading and arithmetic performance during childhood [25], and aerobic fitness has been found to relate to both English/reading and mathematics achievement [9,10]. Interestingly, another study showed specific effects of aerobic exercise training on mathematics achievement, with no benefit to reading, in a sample of 7- to 11-year-old children [49]. Through additional research, we will better understand how both lifestyle factors and neural and cognitive processes account for unique variance in scholastic success, known to forecast future success [26,27]. Mediation models among aerobic fitness, cortical structure, and scholastic success should be tested with larger sample sizes and randomized physical activity interventions to examine whether differences in cortical thickness represent a potential causal pathway between physical activity, fitness, and elevated cognitive and scholastic performance.

The present study arrives at an important time. Physical activity opportunities during the school day are being reduced or eliminated in response to mandates for increased academic classroom time [50], and rising rates of physical inactivity [51–52]. Here we provide additional evidence that increased aerobic fitness levels may enhance cognitive and brain plasticity, with potentially significant outcomes related to scholastic achievement. Additionally, we suggest neural predictors of academic performance. Understanding individual differences in brain health and academic performance has significant implications for educators and policy makers who aim to determine strategies and interventions to maximize learning and success across the lifespan.

## Supporting Information

S1 Dataset Aerobic fitness and cortical thickness data for 48 participants. (SAV)
