# Correction: The Role of Aerobic Fitness in Cortical Thickness and Mathematics Achievement in Preadolescent Children

**DOI:** 10.1371/journal.pone.0138166

**Published:** 2015-09-10

**Authors:** Laura Chaddock-Heyman, Kirk I. Erickson, Caitlin Kienzler, Matthew King, Matthew B. Pontifex, Lauren B. Raine, Charles H. Hillman, Arthur F. Kramer

Fig 1 is incorrect. The authors have provided a corrected version here. The publisher apologizes for the error.

**Fig 1:**
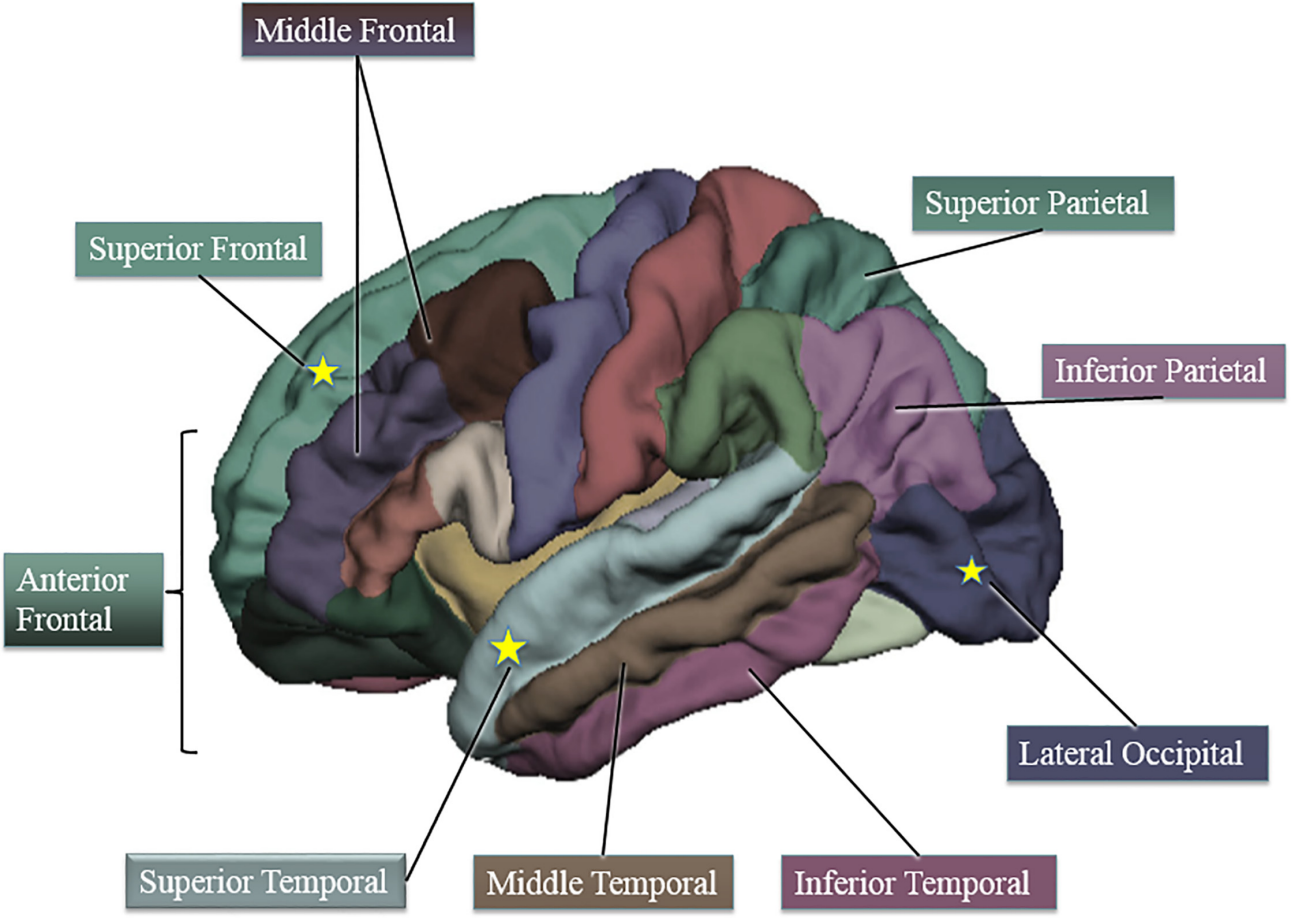
Cortical thickness regions of interest via Freesurfer (adapted from 43). Starred regions are areas in which higher fit children showed decreased cortical thickness compared to lower fit children.
